# The Impact of COVID-19 on Hospitalised COPD Exacerbations in Malta

**DOI:** 10.1155/2021/5533123

**Published:** 2021-06-23

**Authors:** Yvette Farrugia, Bernard Paul Spiteri Meilak, Neil Grech, Rachelle Asciak, Liberato Camilleri, Stephen Montefort, Christopher Zammit

**Affiliations:** Mater Dei Hospital, Triq id-Donaturi tad-Demm, Msida MSD2090, Malta

## Abstract

**Method:**

Data was collected retrospectively from electronic hospital records during the periods 1st March until 10th May in 2019 and 2020.

**Results:**

There was a marked decrease in AECOPD admissions in 2020, with a 54.2% drop in admissions (*n* = 119 in 2020 vs. *n* = 259 in 2019). There was no significant difference in patient demographics or medical comorbidities. In 2020, there was a significantly lower number of patients with AECOPD who received nebulised medications during admission (60.4% in 2020 vs. 84.9% in 2019; *p* ≤ 0.001). There were also significantly lower numbers of AECOPD patients admitted in 2020 who received controlled oxygen *via* venturi masks (69.0% in 2020 vs. 84.5% in 2019; *p* = 0.006). There was a significant increase in inpatient mortality in 2020 (19.3% [*n* = 23] and 8.4% [*n* = 22] for 2020 and 2019, respectively, *p* = 0.003). Year was found to be the best predictor of mortality outcome (*p* = 0.001). The lack of use of SABA pre-admission treatment (*p* = 0.002), active malignancy (*p* = 0.003), and increased length of hospital stay (*p* = 0.046) were also found to be predictors of mortality for AECOPD patients; however, these parameters were unchanged between 2019 and 2020 and therefore could not account for the increase in mortality.

**Conclusions:**

There was a decrease in the number of admissions with AECOPD in 2020 during the COVID-19 pandemic, when compared to 2019. The year 2020 proved to be a significant predictor for inpatient mortality, with a significant increase in mortality in 2020. The decrease in nebuliser and controlled oxygen treatment noted in the study period did not prove to be a significant predictor of mortality when corrected for other variables. Therefore, the difference in mortality cannot be explained with certainty in this retrospective cohort study.

## 1. Introduction

The World Health Organization (WHO) declared COVID-19 as a worldwide pandemic on the 11th of March 2020. Since the first case detected in Wuhan, Hubei Province of China on 31st December 2019, there have been 150.99 million confirmed cases and 3.17 million deaths registered worldwide as of 1^st^ May 2021 [1]. As a result, people's lives have been impacted dramatically, economic growth grinding to a halt, sending the world into a deep recession [2]. The pandemic has also generated a challenge in the routine care of hospitalised patients. Hospital administrations underwent an overhaul with respect to patient management and treatment protocols, aiming to limit the spread of COVID-19 among patients and healthcare workers.

COVID-19 is propagated by the spread of droplets, particularly by aerosol-generating procedures (AGPs) [3]. A conflict of opinion arose among guidelines, where some favoured the use of nebulisers as it was felt that this treatment should not be classified as an AGP, while others recommended abstaining from nebuliser use. In this context, chronic obstructive pulmonary disease (COPD) patients must be considered as they frequently suffer from acute exacerbations requiring hospital admission for treatment including nebulised bronchodilators. Therefore, patients admitted with an acute exacerbation of COPD (AECOPD) are at risk of being unable to access necessary treatment due to a change in guidelines which could have adverse effects on clinical outcomes [4].

During the pandemic, the first COVID-19 case in Malta was confirmed on the 7^th^ of March 2020, imported from Italy [5]. The first cases of local transmission in Malta were later recorded on the 16th of March 2020. As the COVID-19 pandemic began to affect the Maltese Islands, local guidelines were implemented for inpatients judged to have a high risk of carrying the virus. Until these patients were confirmed to test negative for the SARS-CoV-2 virus, the use of nebulised medications, venturi masks, and non-invasive ventilation (NIV) was limited given the risk of aerosol production. Therefore, this led to the alteration of local treatment guidelines for patients who presented with a deterioration in their pre-existing respiratory condition, primarily asthma and COPD, who are the main subgroup of patients who use these treatment modalities.

During the H1N1 pandemic, a study about social behaviour found that even in the early stages of the pandemic, people in Hong Kong started to avoid going to hospital due to a fear of contracting the virus. This fear stemmed from previous experience during the SARS pandemic [6]. In March of 2020, during the first wave of COVID-19, this phenomenon was also demonstrated in Malta, where there was a significant decline in patients presenting with acute cardiac conditions such as myocardial infarctions. A delay in presentation to the Emergency Department (ED) was demonstrated due to a fear of contracting COVID-19 from within the hospital [7]. Containment measures have also impacted hospitalization rates of strokes [8] and myocardial infarctions [9] in centres abroad. A single-centre study in Germany noted a decrease in AECOPD admissions, and the authors postulated that the reasons were multifactorial. The factors discussed include possible COVID-19 protection with COPD treatment, improvement in air quality secondary to lockdown measures, and reduced face-to-face interaction, altogether contributing towards a reduction in AECOPD admissions [10]. Leung et al. (2020) also noted that data for patients presenting with an AECOPD during this pandemic may be skewed because such patients abstain from presenting to hospital due to fear of exposure to SARS-CoV-2, or may seek help late in the disease course, resulting in delayed management and excess mortality in this subgroup [11]. We hypothesised that the decline and delay in presentation of AECOPD admissions noted in other centres was mirrored locally.

## 2. Aim

The aim of this study was to establish the impact of COVID-19 pandemic on the number of AECOPD hospital admissions and their inpatient outcome at Mater Dei Hospital, Malta, between 1st March and 10th May 2020, by comparing to the corresponding period in 2019. Furthermore, we aimed to determine predictors of mortality in AECOPD inpatients.

## 3. Method

This study was a retrospective, observational, cross-sectional study. Data was collected from electronic hospital records. No patients were approached or contacted at any point for the study, and no unique identifying information was collected. The study protocol was approved by the local ethics committee (University of Malta, reference number: FRECMDS_1920_182).

All patients over the age of 18, admitted to Mater Dei Hospital with AECOPD between the 1st of March and 10th of May 2020, were included in this study. The same period from 2019 was used as a control. The diagnosis of an AECOPD was based on the following criteria: change in stable symptoms including increased exertional dyspnoea, chronic cough, and wheeze; the patient's own past medical history and social history, with particular attention to smoking history; imaging; and a response to inhalers and nebulised medications. There were no exclusion criteria. Patients were considered negative for COVID-19 based on results using reverse transcription polymerase chain reaction (RT-PCR).

Assuming a population of 20,000 (4% prevalence of the Maltese population as based on the European Health Interview Survey 2014) and a confidence level of 95%, a sample size of 377 was needed. Statistical analyses were performed using IBM SPSS (SPSS Inc., USA). Variables included gender, locality, medical comorbidities, management modalities used, and inpatient treatment. All variables between the groups were compared using chi-square analysis, Kruskal-Wallis test, and Spearman 2-tailed correlations, accepting a *p* value ≤0.05 as significant. The Fisher exact test was used in cases where sample size was small, such as in the case of non-invasive ventilation use, intensive care admission, and tracheal intubation rate.

To determine predictors of mortality in both cohorts of AECOPD inpatient admissions, a univariate analysis of mortality was carried out using the chi-square test for categorical datasets and independent samples *t*-test for continuous variables.

The major limitation of the chi-square test and independent samples *t*-test is that they investigate the relationship between mortality outcome and a single predictor. It is well known that a lone predictor could be rendered a very important contributor in explaining variations in the mortality outcome but would be rendered unimportant in the presence of other predictors. In other words, the suitability of a predictor in a model fit often depends on what other predictors are included with it. To address this limitation, a logistic regression model was fitted to relate mortality outcome (dependent variable) to the predictors of mortality described above. A forward stepwise procedure was used to identify the parsimonious model.

## 4. Results

A total of 119 and 260 patients were admitted with AECOPD during the period of 1st March to 10th May 2020 and 2019, respectively, demonstrating a 54.2% drop in admissions. There was no significant difference in the mean age of patients (70.9 years in 2020, 71.7 years in 2019; *p* = 0.347). The mean hospital length of stay for patients with AECOPD was 6.76 days (5.89-7.6 days, 95% CI) and 6.74 days (5.48-8.04 days, 95% CI) in 2020 and 2019, respectively (*p* = 0.704). The mean number of previous hospital admissions due to AECOPD per patient was 4.79 (3.48-6.11) and 4.05 (3.32-4.78) in 2020 and 2019, respectively (*p* = 0.148). There were no significant differences between the two study groups with respect to demographics, comorbidities, or COPD pre-admission treatment (Table 1).

**Figure 1:**
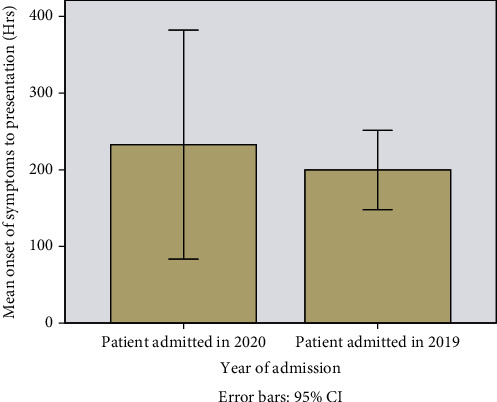
Graph showing the mean time between onsets of symptoms prior to presentation in AECOPD hospitalisations in 2019 compared with 2020.

The mean time from onset of symptoms to hospital admission was longer among patients admitted in 2020, when compared to those admitted in 2019 (232.8 hours (95% CI, 148.08-251.41) vs. 199.7 hours (95% CI, 83.53-382.19)), although this difference did not reach statistical significance (*p* = 0.076) (Figure 1).

**Figure 2:**
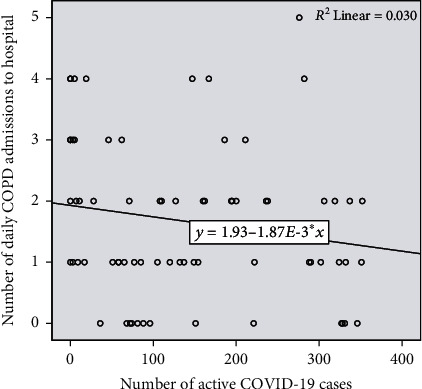
Number of active COVID-19 cases (total and per day) vs. number of AECOPD admissions/day.

A negative correlation was not noted between the number of active COVID-19 cases and AECOPD admissions to hospital (correlation coefficient of -0.208, *R* = 0.03, with a *p* value approaching statistical significance at 0.082) as illustrated in Figure 2.

None of the patients included in the study tested positive for the SARS-CoV-2 virus on RT-PCR testing; however, not all the patients were swabbed. Among the 2020 cohort, 83% (*n* = 99) of patients were screened for the virus, 62% (*n* = 74) were swabbed once, 17% (*n* = 20) were swabbed twice, and 4% (*n* = 5) were swabbed three times. The number of swabs were related to hospital protocols in place at the time which were largely based on the clinical suspicion of COVID-19, the persistence of fever or symptoms despite conventional treatment, or suspicious findings on computed tomography. Patients were kept in isolation until a negative swab was confirmed, as per hospital protocol at the time.

There was a significant reduction in patients who were administered nebulised treatment (salbutamol ± ipratropium bromide) as an inpatient in 2020, when compared to 2019 (60.4% [*n* = 58] vs. 84.9% [*n* = 191]; chi − square = 23.162, *p* ≤ 0.001). In addition, a higher proportion of AECOPD patients in 2020 received supplemental oxygen therapy *via* a normal face mask or a non-rebreather mask during their hospital stay (19% vs. 4.9%, chi − square = 12.024, *p* = 0.002) (Table 2).

No difference in escalation of care was noted between 2020 and 2019 (*p* = 0.457) (Table 3).

**Figure 3:**
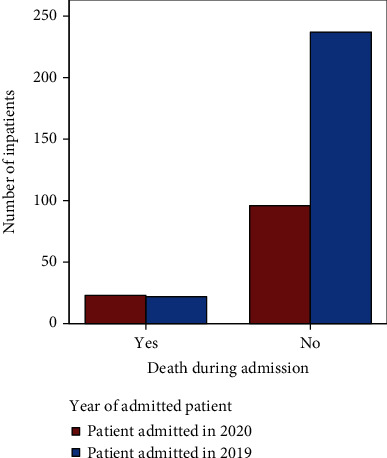
Mortality in 2019 and 2020 during admission.

The proportion of inpatient deaths with an AECOPD was significantly higher in 2020 compared to 2019 (19.3% [*n* = 23] vs. 8.5% [*n* = 22], respectively, chi-square 9.125; *p* = 0.003), as demonstrated in Figure 3.

To determine predictors of mortality, 2020 and 2019 cohorts were both included in a logistic regression analysis. When analysed individually using the chi-square test and the independent samples *t*-test, four continuous variables and nine categorical variables were found to be significantly related to mortality outcome (Tables 4 and 5). Other predictors yielding *p* values larger than the 0.05 criterion were excluded.

To correct for other predictors of mortality, a logistic regression model was fitted to relate mortality outcome to predictors described above (Table 6).

The logistic regression model identifies four significant predictors. Year is the best predictor of mortality outcome and is followed by the lack of use of SABA rescue treatment, active malignancy, and increased length of stay. The Nagelkerke pseudo *R*-square value (0.654) indicates that this 4-predictor parsimonious logistic regression model explains 65.4% of the total variation in the mortality outcome.

## 5. Discussion

In this study, we noted a significant decrease in the number of admissions to Mater Dei Hospital in 2020, compared to 2019. Furthermore, an increased mortality was noted among the 2020 cohort, where the year 2020 was found to be a significant predictor for inpatient mortality. A significant reduction in nebuliser and controlled oxygen use was noted, although this did not significantly impact mortality.

The fall in the AECOPD hospital admission rate between 2019 and 2020 in Malta is likely multifactorial. It is difficult to quantify any single contributing effect and may include potentially improved air quality due to a reduction in air pollution and particulate matter after the closure of businesses and industry and reduced air, sea, and vehicular traffic, increased use of facial masks with a potential resulting decrease in other seasonal respiratory viruses, mandatory shielding of people (including those with respiratory disease, those over 65 years of age, and those on oral corticosteroids and immunosuppressant medication), and fear of presenting to health services due to risk of exposure to the SARS-CoV-2 virus. A similar trend was noted in a study conducted in Hong Kong. Chan (2020) reported that during the first three months of 2020, the admission rate for AECOPD decreased by 44%, compared to the average admission rate in previous years. In this study, the decrease was attributed to masking and increased social distancing [12]. In contrast to Hong Kong, during the period studied, mandatory mask wearing in public was not yet implemented in Malta. The enforcement of mandatory wearing of masks in enclosed public spaces began on the 3rd May 2020 [13]. During this time, people above the age of 65 years and those immunocompromised were encouraged to isolate and to work from home. As of Saturday 17th October 2020, it became mandatory to wear a face mask in indoor and outdoor spaces [14].

It is a well-known fact that pollution contributes heavily to COPD exacerbations, morbidity, and mortality [15]. A study exploring the air quality of the major capital cities worldwide confirmed that with the lockdown measures imposed during the COVID-19 pandemic, there was a reduction in the concentration of PM2.5 (particulate matter less than 2.5 micrometers in diameter) and an improvement in the overall air quality, providing a potential factor for the reduced COPD admissions rate [16]. A study conducted in Bergamo presented evidence that particulate matter may increase transmission of COVID-19. These results may explain why areas having high levels of air pollution also have a higher COVID-19 case concentration [17]. In contrast to expectations, PM2.5 in Malta did not show any reductions following the implementation of COVID-19 measures, unlike nitrogen dioxide (NO_2_), as a major source of PM2.5 is dust from the Sahara Desert [18].

There are various conflicting guidelines regarding whether nebulised treatment is appropriate during the COVID-19 pandemic. NICE and the British Thoracic Society (BTS) recognise that unlike NIV, nebulised medication may not be a viral AGP and does not pose a significant risk for infection with COVID-19 [19, 20]. Contrary, the Global Initiative for Asthma (GINA) advises against nebulised medication use, advocating for the use of pressurised meter dose inhalers (pMDIs) [21].

Between patient groups, a significant decrease in the use of nebulised treatment was noted, as well as a reduction in the administration of controlled oxygen via the venturi mask was noted. This could be explained by fear of hypoxaemia in admitted patients suspected of having the SARS-CoV-2 viral infection. As mentioned above, guidelines concerning the use of nebulised treatment differ widely between academic bodies. NICE advises that as the aerosol from nebulised medication is derived from a non-patient source, it therefore does not carry patient-derived particles and does not carry risk for transmission of COVID-19. There was a shift from controlled oxygen administration to normal face mask and non-rebreather mask supplemental oxygen when comparing 2019 to 2020. This was due to infection control measures implemented at the hospital during the study period limiting the use of venturi masks. Somogyi et al. (2004) demonstrated that venturi oxygen masks can produce a flow of potentially infectious exhaled air during patient expiration [22]. According to Hui et al. (2014), the maximum distances of exhaled air during the application of venturi masks and non-rebreather masks are 0.4 m and <0.1 m, respectively [23]. Following the study period, hospital policies regarding treatment algorithms mentioned above were changed.

This study demonstrated a significant increase in mortality for AECOPD patients admitted during the 2020 COVID-19 pandemic, even though none of these patients tested positive for COVID-19. Based on our results, we hypothesised that lack of controlled oxygen and nebulised treatment would be the predictors towards explaining the difference in mortality. On univariate analysis, thirteen predictors were found to be significant predictors of mortality among all AECOPD inpatient admissions. Among these variables, only differences in oxygen given upon admission and nebulised therapy were significantly different between 2019 and 2020. However, on logistic regression, these two variables did not persist as predictors of mortality. Furthermore, from the predictors found to be significant (active malignancy, length of stay, and SABA pre-admission treatment), none were found to be significantly different between the two cohorts.

Several meta-analyses indicate that COPD patients are at an increased risk of disease severity and mortality if they contract COVID-19 [11, 24–28]. It is therefore recommended that strict adherence to preventer medications is maintained to limit the risk of exacerbations at such a time. The Canadian Thoracic Society highlights this in their updated guidelines [24]. Kaye et al. (2020) found that this recommendation resulted in a general increase in treatment adherence [29]. Of note, during the initial phase of the pandemic, fewer patients attended the outpatient department, resulting in fewer COPD patients being seen by a respiratory specialist. This may have led to poorer control of COPD and contributed to the outcomes.

We cannot therefore explain the difference in mortality found through the results of this study. These results may indicate that the study was relatively underpowered, despite all AECOPD admissions during the study period being included.

## 6. Limitations

All data were collected from online medical records; therefore, the day-to-day ward round notes and treatment charts were not reviewed as these are not available online. A discharge summary was not available for cases where inpatient death occurred, limiting the data available on severity of the AECOPD and treatment received during the hospital stay. Data collection was also limited by the accuracy and completeness of data documented in the discharge summary and online medical records. This study did not evaluate any difference in outpatient mortality in those suffering with COPD, so no comment can be made on any increase in out-of-hospital deaths due to the lack of presentation suggested by the decrease in hospital admissions.

## 7. Conclusions

In conclusion, we noted a statistically significant decrease in hospital admissions of patients with AECOPD during the COVID-19 pandemic in 2020, when compared to 2019. The year 2020 was proved to be a significant predictor for inpatient mortality, with a significantly higher inpatient mortality rate in 2020 compared to 2019. The decrease in nebulised medication administered and controlled oxygen treatment noted in the study period did not prove to be a significant predictor of mortality when corrected for other variables, and we cannot therefore explain with certainty the difference in mortality found in this retrospective cohort study.

## Figures and Tables

**Table 1:** Demographics and comorbidities of patients hospitalised with AECOPD and COPD pre-admission treatment.

Patient demographics	2019		2020		Chi-square	*p* value
	*n*	%	*n*	%		
Gender—male	199	76.5%	89	74.8%	0.137	0.711
Gender—female	61	23.5%	30	25.2%		
Active smoker (within the last 6 months)	102	54.0%	38	43.7%	2.524	0.112
Comorbidity						
Ischaemic heart disease	80	30.8%	28	23.5%	2.028	0.154
Chronic heart failure	103	39.6%	47	39.5%	0.000	0.986
Hypertension	159	61.1%	78	65.5%	0.879	0.349
Diabetes mellitus	71	27.3%	34	28.6%	0.057	0.812
Asthma	17	6.5%	5	4.2%	0.811	0.368
Cerebrovascular disease	22	8.4%	9	7.6%	0.086	0.770
Peripheral vascular disease	19	7.3%	5	4.2%	1.361	0.243
Pulmonary embolism/deep vein thrombosis	11	4.2%	3	2.5%	0.688	0.407
Active malignancy	27	10.4%	18	15.1%	1.771	0.183
Psychiatric history	44	16.9%	23	19.3%	0.257	0.612
COPD pre-admission treatment						
SABA	179	71.0%	90	78.3%	2.108	0.146
LABA	136	54.0%	57	49.6%	0.614	0.433
SAMA	158	62.7%	77	67.0%	0.622	0.430
LAMA	32	12.7%	21	18.3%	1.977	0.160
ICS	122	48.4%	45	39.1%	2.744	0.098
Home nebulisers	21	8.3%	6	5.2%	1.167	0.280
Home NIV	6	2.4%	3	2.6%	0.014	0.906
Home LTOT	64	25.4%	29	25.0%	0.007	0.935

SABA: short-acting beta-agonist; LABA: long-acting beta-agonist; SAMA: short-acting muscarinic antagonist; LAMA: long-acting muscarinic antagonist; ICS: inhaled corticosteroid use; NIV: non-invasive ventilation; LTOT: long-term oxygen therapy.

**Table 2:** Oxygen treatment received in AECOPD inpatients during the study period.

	2019, *n* (%)	2020, *n* (%)	Chi-square	*p* value
Venturi mask used (*n*)	120 (84.5%)	58 (69.0%)	7.541	0.006
Normal face mask/non-rebreather mask (*n*)	7 (4.9%)	16 (19.0%)	11.508	0.001
No oxygen mask (*n*)	15 (10.6%)	10 (11.9%)	0.097	0.756

**Table 3:** Escalation of care of patients hospitalised with AECOPD (analysed using Fisher's exact test).

Escalation of care	2019	2020	Fisher exact test	*Df*	*p* value
NIV (*n*)	24	9	1.589	2	0.457
ICU admission (*n*)	8	6			
Tracheal intubation (*n*)	4	3			

NIV: non-invasive ventilation; ICU: intensive care unit.

**Table 4:** Continuous variables significantly related with inpatient mortality (analysed using independent samples *t*-test).

	Mortality	Sample size	Mean	Standard deviation	*p* value
Patient age on admission (years)	Yes	45	76.71	8.064	<0.001
	No	333	70.75	9.091	

Total number of previous AECOPD admissions (*n*)	Yes	45	1.47	1.791	<0.001
	No	328	4.67	6.644	

Length of stay in hospital (days)	Yes	45	11.73	12.027	0.003
	No	333	6.05	5.717	

Days since last admission (days)	Yes	45	2.07	0.863	0.049
	No	325	1.79	0.888	

**Table 5:** Categorical variables that were significantly related with inpatient mortality (analysed using the chi-square test).

	Death during admission	Alive on discharge	Chi-square	*p* value
Year of admission (2019/2020, *n*)	22/23	237/96	9.125	0.003
Active malignancy (yes/no, *n*)	16/23	29/304	34.289	<0.001
Hypertension (yes/no, *n*)	35/6	201/132	9.803	0.002
Ischaemic heart disease (yes/no, *n*)	17/23	91/242	3.996	0.046
Oxygen given on admission (venturi/normal face mask/no oxygen, *n*)	2/5/1	175/18/24	25.228	<0.001
Nebuliser treatment (yes/no, *n*)	1/4	247/68	9.631	0.002
Admission to ICU (yes/no, *n*)	5/40	9/324	7.859	0.005
SABA pre-admission treatment (yes/no, *n*)	18/16	250/82	7.865	0.005
Diabetes (yes/no, *n*)	18/23	87/246	5.713	0.017

ICU: intensive care unit; SABA: short-acting beta-agonist.

**Table 6:** Predictors of inpatient mortality identified by the logistic regression model.

	Model fitting criteria	Likelihood ratio tests		
	-2 log likelihood of reduced model	Chi-square	*Df*	*p* value
Intercept	14.583	0.000	0	
Year of admission	26.393	11.810	1	0.001
SABA pre-admission treatment	24.360	9.777	1	0.002
Active malignancy	23.580	8.997	1	0.003
Length of stay	18.558	3.975	1	0.046

SABA: short-acting beta-agonist.

## Data Availability

No publicly archived datasets were generated. All available data are included within the manuscript.
